# Acrylamide Neurotoxicity Studies in *Caenorhabditis elegans* Model

**DOI:** 10.3390/antiox14060641

**Published:** 2025-05-27

**Authors:** Zhonglian Ma, Liang Ma, Yuhao Zhang

**Affiliations:** 1 College of Food Science, Southwest University, Chongqing 400715, China; mazhongli1988@163.com (Z.M.); zhy1203@163.com (Y.Z.); 2 College of Agronomy and Life Sciences, Zhaotong University, Zhaotong 657000, China; 3 Yunnan Key Laboratory of Gastrodia and Fungi Symbiotic Biology, Zhaotong University, Zhaotong 657000, China

**Keywords:** acrylamide, neurotoxicity, oxidative stress, *Caenorhabditis elegans*

## Abstract

Acrylamide (ACR), utilized as a precursor for producing polyacrylamide for water purification, has demonstrated neurotoxic properties. However, the mechanisms underlying its neurotoxicity remain inadequately understood. In this investigation, *Caenorhabditis elegans* were exposed to ACR at concentrations ranging from 250 to 1000 μg/mL and then their locomotor behavior, neuronal development, neurotransmitter concentrations, and gene expression profiles were assessed. Exposure to 250–1000 μg/mL ACR resulted in observable behaviors such as head swiveling and body bending, accompanied by a significant reduction in body size. Furthermore, ACR exposure caused damage to serotonergic, cholinergic, dopaminergic, and glutamatergic neuronal structures. In this context, elevated levels of serotonin, dopamine, acetylcholine, and glutamate were detected, along with notable upregulation of the expression of genes associated with neurotransmitters, including *tph-1*, *cat-4*, *mod-1*, *mod-5*, *cat-1*, *ser-1*, *dat-1*, *dop-1*, *dop-3*, *unc-17*, *cho-1*, *eat-4*, and *glr-2*. Moreover, ACR exposure elevated reactive oxygen species (ROS), O_2_, and H_2_O_2_ levels while concurrently depleting glutathione (GSH), thereby compromising the antioxidant defense system. This led to a significant upsurge in the expression of genes involved in the nematode ACR detoxification pathway, specifically *daf-16*, *skn-1*, *mlt-1*, *sod-3*, *gst-4*, *gcs-1*, *hsf-1*, and *hsp-16.2*. Additionally, Spearman correlation analysis revealed a significant inverse relationship between certain neurotransmitter and antioxidant genes and locomotor activities, highlighting the role of these genes in mediating ACR-induced neurotoxicity in *C. elegans*. Collectively, this research enhances the understanding of the mechanisms related to ACR neurotoxicity.

## 1. Introduction

Acrylamide (ACR) is a significant chemical frequently utilized as a binder in various processes, including cement production, water treatment flocculation, and the manufacturing of pesticides. Researchers in Sweden initially documented it as a process contaminant formed during the heating of carbohydrate-rich foods [1]. The global population comes into contact with ACR daily [2,3,4]. ACR is readily generated in starchy items like bread, biscuits, and fried potatoes [5]; additionally, smoking and cooking methods contribute significantly to ACR ingestion [6,7]. The International Agency for Research on Cancer (IARC) classifies ACR as “probably carcinogenic to humans” (category 2A). Notably, ACR can induce hepatotoxic effects by elevating levels of reactive oxygen species (ROS) and influencing the conversion of glutathione and its related enzymes, which subsequently alters several oxidation-related cellular signaling pathways, leading to processes such as apoptosis, inflammation, and autophagy [8]. Furthermore, clinical studies have demonstrated that exposure to ACR can increase both morbidity and mortality rates [2,9,10]. Neurotoxicity is a particular concern linked to ACR exposure; prior studies have proposed that ACR may hinder neurodevelopment via multiple pathways, including oxidative stress, apoptosis, autophagy, and the modulation of the brain–gut axis [11,12,13,14]. The World Health Organisation [15] identifies ACR as one of the chemical hazards for evaluating water quality and emphasizes the need for further investigation regarding ACR exposure.

The neurotoxic impacts of ACR have been documented in both human subjects and animal models. However, the exact mechanisms by which ACR causes neurotoxicity remain largely unclear. Research utilizing simpler genetic models, including rodents and nematodes like *Caenorhabditis elegans*, has provided important insights into its toxicity mechanisms. Toxicological assessments show that prolonged exposure to sublethal levels of ACR impairs the motor function of *C. elegans* [16]. Following exposure to ACR, there is a noticeable reduction in locomotor activities in nematodes, which includes activities such as body bending, head swinging, and swallowing frequency. Furthermore, the observation that even a brief exposure to ACR adversely impacts dopaminergic neurons in *C. elegans* offers potential evidence of neurodegenerative processes induced by ACR [17]. Continuous exposure to ACR results in significant deterioration of dopaminergic and cholinergic neurons in motor-deficient nematodes, whereas GABAergic neurons appear unaffected [18]. Nonetheless, the influence of ACR exposure on other neuronal types associated with motor function remains uncertain and requires additional research.

*C. elegans* serves as a widely utilized model organism known for its precisely characterized nervous system and neuronal development [19,20]. It presents numerous benefits compared to conventional animal models, such as a short lifespan, a fully sequenced genome, well-researched molecular processes, genetic manipulability, ease of culture, and responsiveness to toxic substances. Additionally, its considerable resemblance to mammalian metabolic and neurotransmitter pathways, along with a substantial degree of homology to human genes (ranging from 60% to 80%), including those linked to diseases, renders it an ideal model for studies focusing on neurotoxicity and neuroprotective agents [21,22,23,24,25]. Notably, despite having only 302 neurons, nematodes exhibit a sophisticated variety of behavioral regulatory mechanisms that encompass basic sensory functions, mating behaviors, sleep patterns, drug influences, and even complex learning and memory capabilities [26,27]. Prior research has utilized *C. elegans* to explore neurotoxic effects stemming from microplastics, heavy metals, flame retardants, and biocides, as well as the nervous system’s response to both external and internal harm. The neurotoxicity-related metrics in these nematodes are particularly sensitive markers for gauging the potential toxic impact of various contaminants across multiple assessment criteria [28]. Therefore, *C. elegans* establishes itself as a potent platform for examining the toxicity and mechanisms associated with environmental pollutants, reinforcing its significance as a crucial model for neurotoxicity evaluation.

This study primarily investigated the effects of ACR exposure on motor activity, oxidative damage, neuronal integrity, and neurotransmitter concentrations in *C. elegans*. Additionally, we assessed the expression of genes associated with neurotransmission and the antioxidant response to elucidate the molecular pathways underlying ACR-induced neurotoxicity. Our findings suggest that exposure to ACR may compromise the antioxidant defense system of *C. elegans* by altering neurotransmitter concentrations and gene expression, ultimately resulting in abnormal motor behavior and impaired neuronal development.

## 2. Materials and Methods

### 2.1. Strains, Materials and Regents

The strains used in this investigation comprised the following: wild-type (Bristol N2), BZ555 (dat-1p::GFP), DA1240 (eat-4::GFP), EG1285 (unc-47p::GFP), LX929 (unc-17::GFP), GR1366 [tph-1::GFP + rol-6(su1006)], CF1553 (muIs84[sod-3::GFP]), CL2166 (dvIs19[gst-4::GFP]), and TJ356(zIs356[daf-16::GFP]). All these strains, along with *E. coli* OP50, were sourced from the Caenorhabditis Genetics Center (CGC) located at the University of Minnesota in Minneapolis, MN, USA. The nematode strains were cultured and kept at a temperature of 20 °C on the center of 9 cm nematode growth medium (NGM) plates that were enriched with live *E. coli* OP50 as the food source. Probes including 2,7-dichlorodihydrofluorescein diacetate (DCF), dihydroethidium (DHE), and 10-Acetyl-3,7-dihydroxyphenoxazine (ADHP) were procured from Macklin Biochemical Co., Ltd. (Shanghai, China). BCA protein assay kit was obtained from Beyotime Biotechnology Co., Ltd. (Shanghai, China). Additionally, ACR and various other reagents were supplied by Aladdin Biochemical Technology Co., Ltd. (Shanghai, China).

### 2.2. Experimental Design

Eggs were harvested by lysing gravid adult nematodes with a lysis solution (5% NaOCl/1 N NaOH, ratio of 2:5). Afterward, the eggs underwent washing with K medium and were placed into 12-well plates that contained live *E. coli* OP50, then cultured until they reached the L3 stage, as L3 larvae nematodes exhibit higher sensitivity compared to other developmental stages [29]. A mother liquor of ACR was prepared at a concentration of 10 mg/mL using K solution, which consists of 3.00 g/L NaCl and 2.37 g/L KCl. Synchronized L3 stage *C. elegans* were then transferred to an ACR solution (1 mL) at concentrations of 0, 250, 500, and 1000 μg/mL and exposed for 24 h. The experiment was conducted in 12-well plates (radius of 15.4 mm), with each well containing approximately 100 nematodes and 50 μL of *E. coli* OP50 as a food source, in an incubator (Boxun, Shanghai, China) maintained at 20 °C. Specimens of *C. elegans* were subsequently collected and rinsed with K medium for further evaluation. Three independent experiments were performed.

### 2.3. Body Size Assay and Locomotory Behavior

#### 2.3.1. Body Size Assay

Body measurements concerning the length and width of the nematodes were conducted using a fluorescence microscope (OLYMPUS BX43FC, Evident (Guangzhou) Co., Ltd., China) to evaluate their growth metrics. The lengths and widths were analyzed using ImageJ software version 2.3.0, developed by the National Institutes of Health in Bethesda, MD, USA.

#### 2.3.2. Locomotory Behavior

The assessment of neurobehavioral development was conducted by observing head thrashes and body bends [30]. Nematodes were placed on NGM plates devoid of food, and observations were performed using a stereomicroscope. Following a recovery period of two minutes, we analyzed the varying directions associated with the mid-body bending movements of the worms. Body bends were defined as directional changes related to movement along the *Y*-axis by the posterior bulb during pharyngeal ball activity, while it was assumed that *C. elegans* moved along the *X*-axis. To ensure that the results obtained across the assays were statistically significant, thirty individual nematodes were examined for each assay [31,32].

#### 2.3.3. Foraging Behavior and Chemotaxis Assay

Following methods used in previous studies [33], the food-sensing behavior test was conducted on strain N_2_, which was washed three times with K medium after 24 h of exposure to ACR. Approximately 30 nematodes were transferred to the starting position of the test Petri dish (figure in below), and the number of nematodes contacting the target circle containing food was observed under a microscope after 12 and 24 h, respectively. The predation rate was calculated as the number of nematodes contacting the target circle divided by the total number of nematodes present in the test culture dish.

In the chemotaxis assay [34], nematodes from each group were rinsed with NaCl-free K solution and subsequently transferred to either NaCl-containing or NaCl-free NGM for a starvation period of 3 h. Approximately 30 nematodes were positioned at the starting point on the test Petri dishes (figure in below). Concurrently, a drop of 1% NaN3 was applied at two locations to immobilize the nematodes. After allowing the nematodes to roam freely for 0.5 h, the number of nematodes located within a 1 cm radius of both the NaCl and non-NaCl points was counted.

### 2.4. ADHP, DHE, and NDA Fluorescent Staining of C. elegans

The worms were cultivated according to the procedure outlined in Section 2.2. Afterward, they were rinsed three times with K medium. The levels of H_2_O_2_ production, O_2_^−^ production, and reduced glutathione (GSH) were assessed using ADHP (50 μM for 2 h), DHE (8 μM for 2 h), and NDA (40 μM for 2.5 h) as detailed by [35]. Following this, the dyes were removed, and worms were randomly selected for fluorescence microscopy observation during the equivalent exposure period.

### 2.5. Levels of Endogenous ROS and Accumulation of Lipofuscin in Nematodes

Endogenous levels of ROS in nematodes were assessed through a modified H_2_DCF-DA assay [36]. In brief, after a 24 h supplementation with ACR, the worms were treated with 10 μM H_2_DCF-DA in K medium for 2 h at 20 °C in darkness. The worms were then observed and photographed using a fluorescence microscope (OLYMPUS BX43FC, Evident (Guangzhou) Co., Ltd., China), with Image J software employed for analyzing the average fluorescence intensity and quantifying the extent of ROS accumulation in the nematodes.

### 2.6. Morphological Changes of Neurotransmitter Systems

Strains BZ555, EG1285, GR1366, LX929, and DA1240 were utilized to assess neurons classified as dopaminergic, GABAergic, serotonergic, cholinergic, and glutamatergic, respectively. The nematodes were cultured according to the procedures outlined in Section 2.2. A total of three independent trials were conducted, with measurements taken from 30 worms at each concentration. An inverted fluorescence microscope (OLYMPUS BX43FC, Evident (Guangzhou) Co., Ltd., China) was employed to capture fluorescence images of thirty nematodes from each group. Furthermore, the fluorescence intensity of GFP was quantified using ImageJ software. Each transgenic nematode was photographed under standardized conditions.

### 2.7. Neurotransmitter Levels

Nematodes exposed to experimental conditions were collected and preserved at −20 °C for subsequent analyses. The levels of neurotransmitters, including GABA and serotonin (Jianglai Biology, Shanghai, China), and dopamine, glutamate, and acetylcholine were assessed using enzyme-linked immunosorbent assay (ELISA) kits (Yuanju Biotechnology, Shanghai, China), adhering to the manufacturer’s instructions, and normalized relative to the corresponding protein content.

### 2.8. DAF-16::GFP Localization and SOD 3::GFP and GST-4::GFP Quantification

Worms that did not undergo ACR treatment served as the control group. The localization of the DAF-16 protein, tagged with a green fluorescent protein (GFP), in the transgenic strain TJ356 was examined with a fluorescent microscope (OLYMPUS BX43FC, Evident (Guangzhou) Co., Ltd., China). The fluorescent signal accumulation within the nuclei was assessed. The activity of SOD-3::GFP and GST-4::GFP was evaluated using the transgenic strains CF1553 and CL2166. GFP expression levels were quantified through Image J software, analyzing at least 30 worms for each experimental group.

### 2.9. qRT–PCR Analysis

RNA was extracted from the treated specimens utilizing an RNA extraction kit (Tiangen, Beijing, China) according to the manufacturer’s protocols. The extracted RNA then underwent a reverse transcriptase reaction with a reverse transcriptase kit (Vazyme Biotech, Nanjing, Jiangsu, China), leading to the synthesis of complementary DNA (cDNA). Furthermore, quantitative reverse transcription PCR (qRT-PCR) was employed to assess relative gene expression, with act-1 used as an internal control, using a Step One Plus RT-PCR system (Applied Biosystems, Singapore). The 2^−(ΔΔCt)^ method was applied to determine the relative mRNA levels. A minimum of three replicates for each reaction was performed in every experiment. The specific methods and the primer sequences designed for qRT-PCR can be found in Table S1. 

### 2.10. Statistical Analysis

Statistical analyses were performed using SPSS Statistics 24.0 software. Data are presented as means ± standard errors of the mean (SEMs). Differences among groups were assessed using one-way analysis of variance. Statistical significance is indicated in the figures as follows: ns (not significant), * *p* < 0.05, ** *p* < 0.01, and *** *p* < 0.001.

## 3. Results

### 3.1. Dose-Dependent Impairment of Fitness by ACR in C. elegans

**Figure 1:**
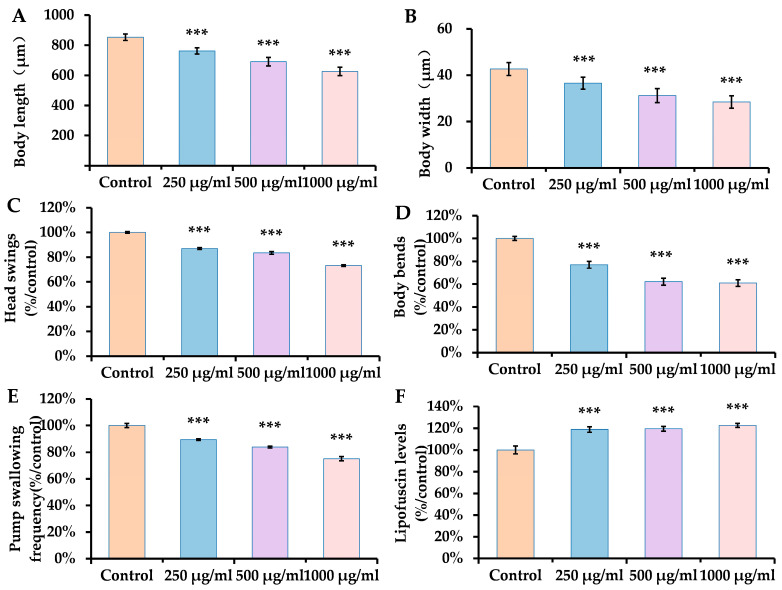
Effect of ACR on body length (**A**), width (**B**), head swinging (**C**), body bending (**D**), swallowing frequency (**E**) and lipofuscin accumulation (**F**). Results are presented as means ± SEMs (*n* = 30 replicates per group) in comparison to the control group. Statistical significance is indicated in the figures as follows: *** *p* < 0.001.

The phenotypic characteristics of worms fed with ACR were assessed using several typical biomarkers. These included body size, motility, and lipofuscin accumulation levels. The length and width of bodies serve as crucial metrics for evaluating nematode growth and can also act as indicators of toxicity relating to their development. In this study, we observed a decrease in nematode body length that corresponded with rising concentrations of ACR. As illustrated in Figure 1A,B, ACR markedly reduced both body length and width at concentrations of 250, 500, and 1000 μg/mL, showing reductions ranging from 10.70% to 26.64% for length and 14.33% to 33.41% for width, in comparison to the control group. Our findings indicate that ACR adversely affects the growth and development of nematodes.

*C. elegans* possesses a total of 69 motor neurons and 95 muscle cells, which are crucial for various movement patterns [37]. The head swing represents an active locomotion state in nematodes, influencing their perception and decision-making processes [38]. Enhanced levels of ACR induced a gradual decline in the frequency of head swings, revealing a noteworthy reduction of 12.78% to 26.72% in nematodes subjected to ACR concentrations of 250, 500, and 1000 μg/mL compared to the control group, indicating that ACR diminishes the nematodes’ environmental perception capabilities. The abdominal and dorsal muscles of the nematode contract and relax alternately, culminating in a smooth crawling movement characterized by a sinusoidal pattern [37]. This experiment involved an assessment of muscle function. With rising concentrations of ACR, a significant decline in the frequency of body bends in worms was observed (Figure 1C,D), exhibiting a marked reduction in flexion frequency ranging from 22.99% to 39.08% compared to the control group. This suggests that ACR could potentially impair muscle function, resulting in sluggish locomotor activity. 

*C. elegans* utilizes rhythmic contractions of its pharyngeal muscles, known as pharyngeal pumps, to filter, transport, and grind food particles [39]. In this investigation, the swallowing frequency in *C. elegans* diminished considerably with increasing concentrations of ACR, showing a reduction of 10.41% to 24.87% compared to the control group. This implies that ACR may negatively affect either the pharyngeal muscles or associated nerves, thereby interfering with food intake and retarding growth.

Furthermore, lipofuscin accumulation is a recognized marker of senescence. As can be seen in Figure 1F, lipofuscin was significantly increased by 18.85–22.52% in ACR 250, 500, and 1000 μg/mL-exposed *C. elegans* as compared to the control group.

### 3.2. Dose-Dependent Impairment of Oxidative by ACR in C. elegans

**Figure 2:**
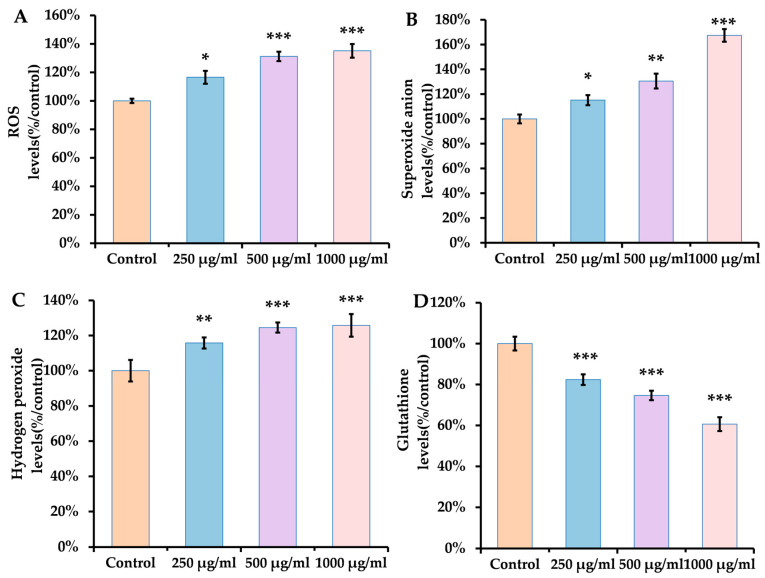
Effect of ACR on (**A**) ROS, (**B**) O_2_^−^, (**C**) H_2_O_2_, and (**D**) GSH. Results are presented as means ± SEMs (*n* = 30 for each group) in comparison to the control group. Statistical significance is indicated in the figures as follows: * *p* < 0.05, ** *p* < 0.01, and *** *p* < 0.001.

The levels of O_2_^−^, H_2_O_2_, and ROS are used as markers for evaluating oxidative stress [40]. Glutathione (GSH) is recognized as a highly effective intracellular antioxidant that directly interacts with active electrophiles, helping to sustain the cellular redox balance [41]. Studies have indicated that ACR exposure may cause an elevation in O_2_^−^ levels and a reduction in GSH [35,42]. In our investigation, we utilized DCF, DHE, ADHP, and NDA probes to measure the concentrations of ROS, O_2_^−^, H_2_O_2_, and GSH in N_2_. The fluorescence quantification results depicted in Figure 2A–C reveal a marked increase in fluorescence intensity in the group treated with ACR, demonstrating a dose-dependent relationship. The NDA fluorescent dye interacts with GSH, producing green fluorescence. Furthermore, Figure 2D illustrates a decline in GSH levels within the group subjected to ACE treatment. These findings indicate that ACE exposure disrupts the antioxidant defense mechanism by elevating ROS, O_2_^−^ and H_2_O_2_ levels while leading to a depletion of GSH.

### 3.3. Dose-Dependent Impairment of Sensory Behavior by ACR in C. elegans

**Figure 3:**
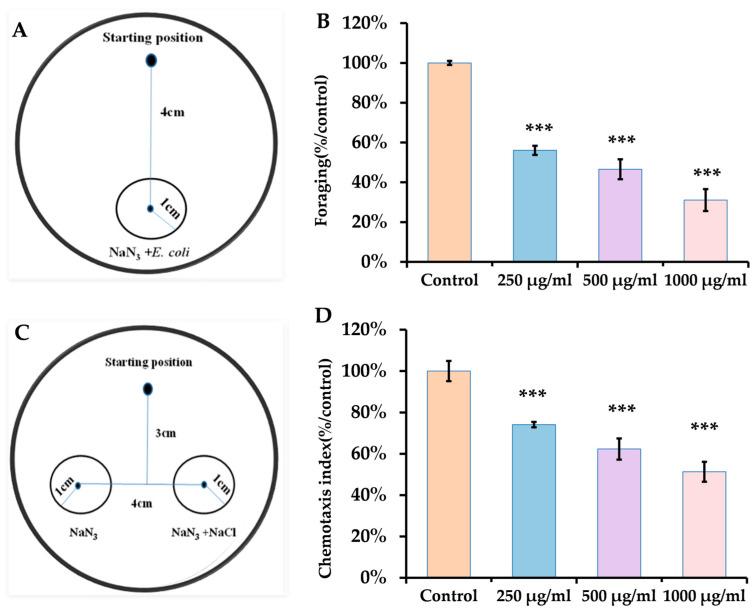
Effect of ACR on the foraging behavior and chemotaxis index. (**A**). Schematic diagram of a test dish for the feeding behavior of *C. elegans*. (**B**). Foraging behavior. (**C**). Schematic diagram of a test dish for chemotaxis index of *C. elegans*. (**D**). Chemotaxis index. Data are presented as means ± SEMs (*n* = 30 for each group) in comparison to the control group. Statistical significance is indicated in the figures as follows: *** *p* < 0.001.

Upon exposure to ACR at concentrations of 250, 500, and 1000 μg/mL for 24 h, there was a significant alteration in the foraging behavior of nematodes, resulting in reductions of 43.93%, 53.44%, and 68.91% for each respective treatment when compared to the control group (Figure 3B).

*C. elegans* employs both chemical and olfactory receptors to detect environmental changes such as odors and temperature [43]. In response to various chemicals’ odors, they modify their behavior by either moving toward or avoiding these stimuli [44]. The organism can be drawn to concentrations of NaCl ranging from 0.1 to 200 mM through NaCl-sensing neurons [45]. Analyzing the preference of nematodes for NaCl across different treatment groups revealed that the chemotaxis index for those exposed to ACR was notably reduced compared to the control group, and this index decreased further with rising ACR concentrations (Figure 3D). This indicates that ACR impacts the NaCl-sensing neurons of the nematodes, ultimately diminishing their capacity to perceive the NaCl odor.

### 3.4. Dose-Dependent Impairment of Neural by ACR in C. elegans

**Figure 4:**
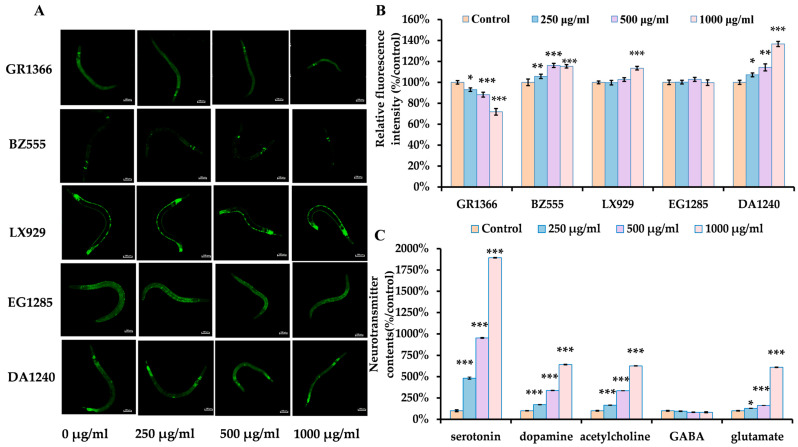
Effect of ACR on GR1366 (tph-1::GFP), BZ555 (dat-1::GFP), LX929 (unc-17::GFP), EG1285 (unc-47::GFP), DA1240 (eat-4::GFP) and neurotransmitters. (**A**) Fluorescent images depicting five neurons from nematodes. (**B**) Relative fluorescence intensity in five neurons in nematodes. (**C**) The levels of GABA, serotonin, dopamine, acetylcholine, and glutamate in comparison to the control group. Statistical significance is indicated in the figures as follows: * *p* < 0.05, ** *p* < 0.01, and *** *p* < 0.001.

The impact of ACR exposure on the dopaminergic, glutamatergic, GABAergic, serotonergic, and cholinergic neurons of nematodes was assessed. The nervous system plays a crucial role in managing and regulating the motor behaviors of *C. elegans*, with neuronal function significantly influenced by neuronal development. Figure 4A,B illustrate the morphological alterations of these five neuron types alongside their respective fluorescent intensities in the worms. Serotonergic neurons were identified using GFP driven by tph-1::GFP in the GR1366 strain, where ACR exposure resulted in a notable decline in fluorescence intensity within these serotonergic neurons compared to the control group (*p* < 0.05). Additionally, dopaminergic neurons in the BZ555 strain were marked with the transporter marker dat-1::GFP (Figure 4A), while the DA1240 glutamatergic neurons were tagged with the transporter marker eat-4::GFP. Following 24 h of ACR treatment at concentrations of 250, 500, and 1000 µg/mL, an increase in the number of fluorescent dots was observed in both BZ555 dopaminergic and DA1240 glutamatergic neurons. Quantitative analysis demonstrated that the relative fluorescence intensities in the BZ555 and DA1240 strains increased significantly by approximately 5.72–16.16% and 7.17–36.64%, respectively, compared to the control groups. Cholinergic neurons in the LX929 strain were detected using GFP driven by unc-17::GFP; however, no significant structural changes in GABA neurons or variations in relative fluorescence intensity were observed after exposure to varying ACR concentrations over the examined 24 h period.

Neurons are especially vulnerable to oxidative stress, which subsequently influences the synthesis of neurotransmitters among neurons [46]. Consequently, to explore the neurotoxic effects of ACR on nematodes, researchers examined the impacts of neurotransmitters such as GABA, serotonin, dopamine, acetylcholine, and glutamate in nematodes subjected to ACR exposure. Figure 4C illustrates that, after 24 h of exposure to ACR concentrations of 250, 500, and 1000 μg/mL, there were notable increases in the levels of serotonin, dopamine, acetylcholine, and glutamate compared to the control group, with percentage changes of 383.12% to 1794.22% (*p* < 0.001), 71.92% to 541.55% (*p* < 0.001), 65.69% to 526.36% (*p* < 0.001), and 28.49% to 509.88% (*p* < 0.05), respectively.

### 3.5. ACR Changes the Expression of Genes Related to Neurotransmitters and Antioxidant Function in C. elegans

**Figure 5:**
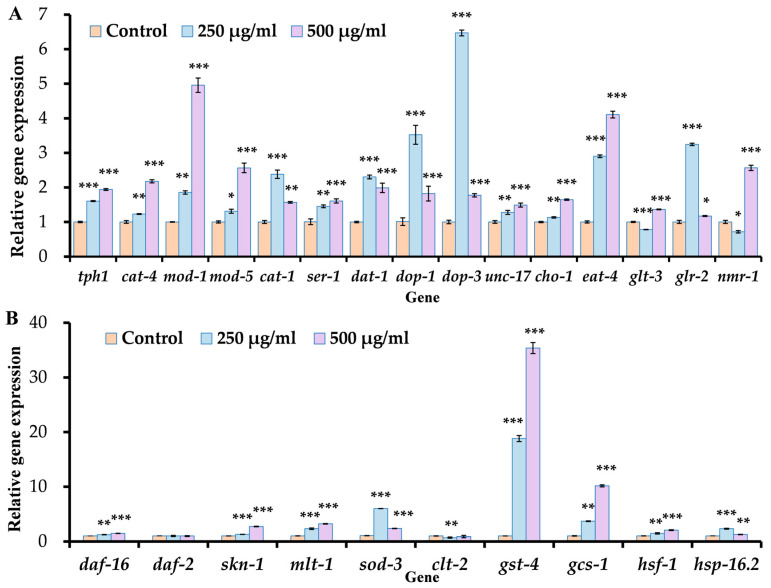
(**A**) Effects of ACR on the mRNA expression levels of genes associated with serotonin, dopamine, acetylcholine, and glutamate in *C. elegans*. (**B**) Effects of ACR on the mRNA levels of genes related to antioxidant function in *C. elegans*. Data are normalized by the expression of act-1 and are presented as means ± SEMs (*n* = 3) compared to the control group. Statistical significance is indicated in the figures as follows: * *p* < 0.05, ** *p* < 0.01, and *** *p* < 0.001.

To further clarify the effects of ACE exposure on the neurotransmitters serotonin, dopamine, acetylcholine, and glutamate, we selected 250 μg/mL and 500 μg/mL concentrations of ACR for qRT-PCR analysis to assess the mRNA levels of 15 genes linked to these four neurotransmitters. As illustrated in Figure 5A, a 24 h exposure to ACE resulted in a significant increase in the expression of the *tph-1*, *cat-4*, *mod-1*, *mod-5*, *cat-1*, *ser-1*, *dat-1*, *dop-1*, *dop-3*, *unc-17*, *cho-1*, *eat-4*, and *glr-2* genes when compared to the control group. The data demonstrated a notable upregulation in the expression levels of genes related to neurotransmitter transport and receptor binding in *C. elegans* after 24 h of ACE exposure. Specifically, the levels of *tph-1* increased by 1.61–1.94-fold, *cat-4* by 1.23–2.18-fold, *mod-1* by 1.85–4.96-fold, *mod-5* by 1.31–2.57-fold, *cat-1* by 1.57–2.38-fold, *ser-1* by 1.45–1.61-fold, *dat-1* by 1.99–2.30-fold, *dop-1* by 1.82–3.52-fold, *dop-3* by 1.77–6.47-fold, *cho-1* by 1.13–1.65-fold, *eat-4* by 2.90–4.11-fold, and *glr-2* by 1.17–3.24-fold, illustrating marked differences from the unexposed group. Additionally, the mRNA levels of *tph-1*, *cat-4*, *mod-1*, *mod-5*, *ser-1*, *unc-17*, *cho-1*, and *eat-4* demonstrated a dose-dependent response following ACR exposure in *C. elegans*. These results suggest that ACR exposure triggers an upregulation in the expression of genes related to serotonin, dopamine, acetylcholine, and glutamate. The genes associated with these neurotransmitters are essential in managing their synthesis, transport, release, and overall signaling.

Increased ROS levels and activation of associated free oxygen radicals can promote neuronal degeneration [47]. In order to explore the potential mechanisms underlying the neuronal damage induced by ACR in nematodes, we assessed the expression levels of genes related to antioxidants through quantitative real-time PCR assays (Figure 5B). ACR treatment resulted in a significant increase (*p* < 0.01) in the expression of *daf-16*, *skn-1*, *mlt-1*, *sod-3*, *gst-4*, *gcs-1*, *hsf-1*, and *hsp-16.2* compared to the control group. The expression changes were recorded as follows: *daf-16* increased by 1.22–1.46 fold, *skn-1* increased by 1.28–2.71 fold, *mlt-1* by 2.32–3.20 fold, *sod-3* by 2.40–6.01 fold, *gst-4* by 18.81–35.38 fold, *gcs-1* by 3.69–10.16 fold, and *hsp-16.2* by 1.25–2.31 fold. In contrast, *ctl-2* demonstrated a reduction of approximately 11.38% to 29.74%. These results suggest that the DAF-16/FOXO pathway and SKN-1/Nrf2 may mediate the antioxidant mechanisms associated with ACR.

### 3.6. ACR Exposure Increases DAF-16 and the Expression of SOD-3 and GST-4

**Figure 6:**
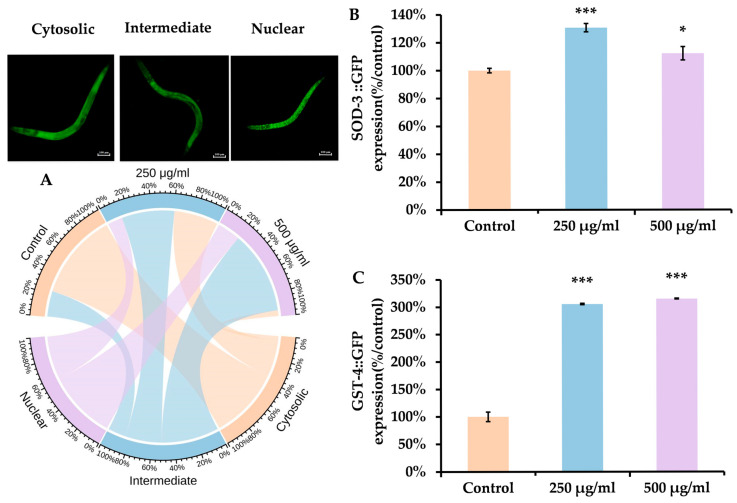
(**A**) The locations of DAF-16::GFP in the ‘cytosolic’, ‘intermediate’, and ‘nuclear’ regions. (**B**) Impact of ACR on SOD-3::GFP and GST::GFP. (**C**) The data are presented as means ± SEMs (*n* = 30 for each group). * *p* < 0.05, and *** *p* < 0.001, in comparison to the control group.

Exposure to ACR induces various effects on oxidative stress. To understand the molecular pathways through which ACR causes neurotoxicity in nematodes, we examined the impact of ACR on the transcriptional activity of the transcription factor DAF-16. The downstream factors controlled by DAF-16 regulate a range of factors related to lifespan, stress response, metabolism, and proteolysis, making them prime candidates for antioxidant and neuroprotective investigations [48]. When DAF-16 is activated, there is an upregulation of the expression of antioxidant enzymes like superoxide dismutase (SOD), which plays a critical role in safeguarding cells against oxidative injury. As illustrated in Figure 6A, ACR significantly enhanced the nuclear localization of DAF-16 (Figure 6A; *p* < 0.001). The expression levels of SOD-3 and GST-4 were assessed using the CF1553 and CL2166 mutant strains (Figure 6B,C), given their significance as downstream targets of DAF-16. Compared to the control, fluorescence intensity after ACR exposure demonstrated a marked increase, signifying elevated levels of SOD-3 and GST-4 expression. These findings suggest that ACR modulates the activities of DAF-16.

### 3.7. Spearman’s Correlation Analysis

**Figure 7:**
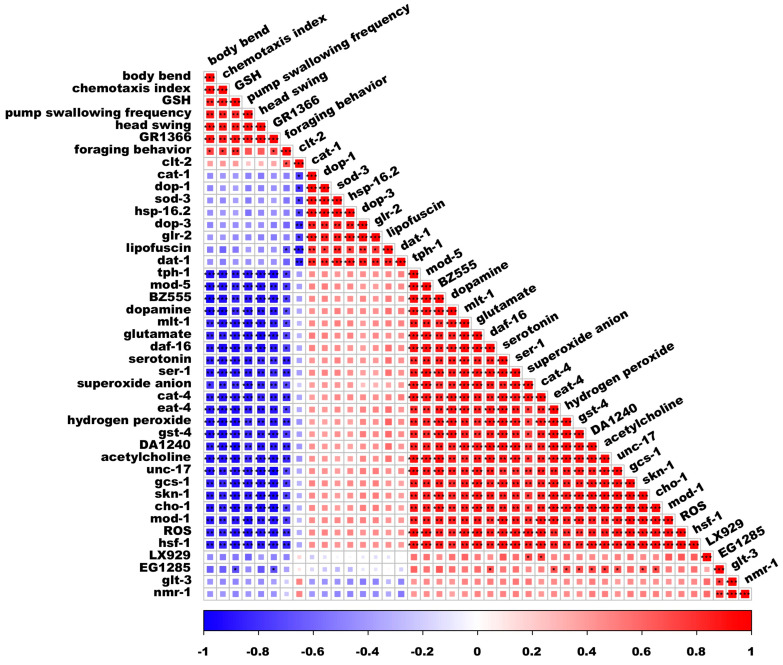
Spearman’s correlation analysis. * *p* < 0.05, ** *p* < 0.01, and *** *p* < 0.001.

The relationship between neurotoxicity and oxidative stress, as well as neurotransmitters, was assessed using Spearman’s correlation analysis (Figure 7). Following a 24 h treatment with ACR, levels of GSH showed significant positive correlations with a minimum of three behavioral metrics. Specifically, GSH correlated with body bending (*p* < 0.01), the frequency of pump swallowing (*p* < 0.01), and foraging behavior (*p* < 0.01).

Conversely, the levels of four neurotransmitters—dopamine, glutamate, serotonin, and acetylcholine—were found to have significant negative correlations with at least three behavioral indicators. For instance, dopamine demonstrated a correlation with body bending (*p* < 0.001), the chemotaxis index (*p* < 0.001), and foraging behavior (*p* < 0.05). Similarly, glutamate was linked to body bending (*p* < 0.01), the chemotaxis index (*p* < 0.01), pump swallowing frequency (*p* < 0.001), head swings (*p* < 0.001), and foraging behavior (*p* < 0.05). Moreover, serotonin correlated with body bending (*p* < 0.01), pump swallowing frequency (*p* < 0.01), head swings (*p* < 0.01), and foraging behavior (*p* < 0.01), while acetylcholine was associated with body bending (*p* < 0.001), pump swallowing frequency (*p* < 0.01), and foraging behavior (*p* < 0.01). The genes *mph-1*, *mod-5*, *ser-1*, *cat-4*, *eat-4*, *unc-17*, *cho-1*, and *mod-1*, which are related to serotonin, dopamine, acetylcholine, and glutamate, showed significant negative correlations with at least three behavioral indicators.

The genes associated with oxidative stress, including *daf-16*, *gst-4*, *gcs-1*, *hsf-1*, and *skn-1*, exhibited a significant negative correlation with a minimum of four behavioral indicators. Additionally, ROS, superoxide anion, and hydrogen peroxide demonstrated a highly significant negative relationship with at least three behavioral indicators in *C. elegans*. These findings indicate that exposure to ACR may diminish the fitness of nematodes and could be linked to *daf-16* and *skn-1*.

## 4. Discussion

ACR is a commonly encountered chemical compound that has shown increased human exposure, particularly through food sources [13], and it has the capability to cross the blood–brain barrier, resulting in neurotoxic effects [49]. This study aimed to examine the impact of ACR on the locomotion of *C. elegans* and to investigate its potential neurotoxicological mechanisms. Following exposure to ACR, the growth and development (including body length and width) of *C. elegans* were found to be inhibited when compared to a control group. Additionally, our findings revealed that ACR exposure significantly decreased the head sway of *C. elegans*, and a reduction in pump contractions may hinder the growth and developmental processes of the nematodes, as these contractions may be indicative of their feeding frequency. A lower feeding frequency could lead to growth delays. The results show a marked reduction in both the foraging ability and chemotaxis index of *C. elegans* subjected to ACR compared to the unexposed group. Research indicates that nematodes can accurately detect food sources through intricate interactions among various neurons [33,50], and tests measuring the chemotaxis index demonstrate the behavioral adaptability of these nematodes [51]. The assessment of neurotoxicity in *C. elegans* is based on motor behavior [52]; deficits in behavior due to ACR exposure may also signal the presence of neurobehavioral toxicity, which appears to be positively correlated with the dosage of exposure.

The development of neurons plays a pivotal role in determining neuronal function across various organisms [53,54]. We further examined the effects of ACR on neurons that are serotoninergic, dopaminergic, cholinergic, glutamatergic, and GABAergic in nature. ACR has the potential to inflict considerable damage on the GFP expression markers BZ555 (dat-1::GFP) and LX929 (unc-17::GFP). Our findings align with existing research that utilized GFP expression levels as metrics for assessing neurodegenerative conditions [18]. Additionally, this investigation revealed that ACR adversely affected the GFP expression markers GR1366 (tph-1::GFP) and DA1240 (eat-4::GFP). Furthermore, Spearman’s correlation analysis indicated a significant relationship between the fluorescence intensity of dopaminergic and glutamatergic neurons in the BZ555 and DA1240 strains and the motility behavior of the nematodes. This suggests that the neurobehavioral toxicity of ACR in nematodes may be intricately linked to the degeneration of dopaminergic and glutamatergic neurons. In nematodes, the signaling functions of dopaminergic and glutamatergic neurons are crucial neurobehavioral elements associated with motility. Dopaminergic neurons exhibit a high degree of dynamism and can be influenced by external environmental factors, ultimately affecting the crawling locomotion of nematodes [55]. Glutamatergic neurons consist of 16 distinct types, all participating in the locomotion of nematodes [56]. This research indicates that the function of neuronal systems in nematodes may be compromised since exposure to ACR leads to diminished fluorescence intensity in serotonergic neurons; heightened luminescence in glutamatergic, dopaminergic, and cholinergic neurons; and escalated neurodegeneration. Similarly, a previous investigation observed considerable degeneration of dopaminergic and cholinergic motor neurons in nematodes subjected to ACR, which resulted in motor impairments, while GABAergic motor neurons remained unaffected [18]. Our results align with these observations under the conditions of our experiments. Moreover, neurotransmitters play a vital role in conveying excitatory or inhibitory signals between neurons, being released from activated neurons and communicated to target cells at the synapse [57]. Variations in neurotransmitter levels are also valuable indicators for investigating the toxicological impacts of chemicals within the central nervous system [58]. Exposure to ACR markedly enhances the levels of dopamine, serotonin, glutamate, and acetylcholine (Figure 4C). Consequently, ACR not only inflicts harm on the nervous system (Figure 4A) but also disrupts the normal neurotransmission of serotonin, dopamine, acetylcholine, and glutamate, potentially offering new insights into the mechanisms of neurobehavioral toxicity associated with ACR. In summary, ACR exposure impacts the development of nematode neurons and modifies neurotransmitter levels, emphasizing its neurotoxic consequences.

To evaluate the impact of neurotransmitters on locomotor behavior, foraging, and chemotaxis, a quantitative reverse transcription polymerase chain reaction (qRT-PCR) analysis was conducted to investigate transcriptional variations in numerous genes associated with serotonin, dopamine, acetylcholine, and glutamate. Serotonin plays a crucial role in regulating feeding behaviors across various nematode species; it stimulates the pharynx, enhances the pharyngeal pumping rate, and modulates both feeding and locomotor activities in nematodes [59]. Furthermore, serotonin significantly influences the development of feeding behaviors within the nematode phylum. The *tph-1* gene codes for tryptophan hydroxylase [60], while the *mod-1* gene encodes an ionotropic serotonin transporter [61]. Additionally, the *cat-1* gene encodes a vesicular transporter essential for incorporating serotonin into synaptic vesicles, and *mod-5* is the sole serotonin reuptake transporter [62]. This investigation revealed that mRNA expression levels of the genes *tph-1*, *mod-1*, *mod-5*, *cat-4*, and *cat-1* significantly increase when exposed to ACR at concentrations of 250 and 500 μg/mL. The significant modifications observed in these genes suggest that ACR exposure not only affects serotonin uptake but also promotes serotonin synthesis and transport to a certain degree. Moreover, the genes *dop-1* and *dop-3* encode dopamine receptors, with the *dat-1* gene coding for the presynaptic dopamine transporter that governs dopamine synthesis and reuptake [63]. A recent investigation revealed that these genes are crucial for modulating learned avoidance behavior in nematodes [64]. Similarly, Yu et al. discovered that exposure to carboxyl-modified polystyrene microplastics influenced the expression of these genes, resulting in neurobehavioral deficits. The *eat-4* gene is responsible for encoding a glutamate transporter, while the *glr-2* gene encodes a receptor for glutamate [65]. Notably, *cho-1* is a gene that codes for a high-affinity choline transporter, which facilitates the transport of choline to the presynapse for acetylcholine synthesis [66], whereas *unc-17* encodes a transporter that moves acetylcholine from the presynapse to the synapse [67]. Our findings indicated that exposure to ACR leads to a marked increase in synaptic acetylcholine levels (Figure 4C). This effect is primarily achieved through the modulation of nicotinic acetylcholine receptor activity, which enhances neurotransmission, as a result of the upregulation of genes such as *cho-1* and *unc-17*. Furthermore, Spearman’s correlation analysis indicated significant negative correlations for the genes *tph-1*, *mod-5*, *ser-1*, *cat-4*, *eat-4*, *unc-17*, *cho-1*, and *mod-1*, which are associated with serotonin, dopamine, acetylcholine, and glutamate, with at least three behavioral metrics. In summary, exposure to ACR alters the neurotransmission processes of serotonin, dopamine, acetylcholine, and glutamate by modifying the expression of genes including *cho-1*, *unc-17*, *dat-1*, *dop-1*, *dop-3*, *mod-1*, *mod-5*, and *tph-1*, leading to neurotoxic effects in nematodes.

Furthermore, ROS are defined as compounds containing oxygen that are generated during aerobic metabolism, including peroxides, superoxide anions, and hydroxyl radicals. When the levels of ROS surpass the organism’s antioxidant defense mechanisms, oxidative stress may occur, potentially harming cellular structures and resulting in cellular dysfunction, which can adversely affect lipids, proteins, and DNA [68,69]. This mechanism could be harmful. In our experiment, we also observed that *C. elegans* exposed to ACR experienced an increase in ROS production, specifically O_2_^−^, and H_2_O_2_, suggesting that ACR might enhance ROS generation in *C. elegans*, thereby influencing the expression of other genes associated with oxidative stress. Our findings indicate that ACR notably raised the expression levels of several genes (*daf-16*, *skn-1*, *mlt-1*, *sod-3*, *gst-4*, *gcs-1*, *hsf-1*, and *hsp-16.2*) linked to both the DAF-16/FOXO pathway and the SKN-1/Nrf2 signaling pathway. Spearman’s correlation analysis indicated that the genes *daf-16*, *gst-4*, *gcs-1*, *hsf-1*, and *skn-1* exhibited significant negative correlations with at least four behavioral indicators. Additionally, ROS, superoxide anions, and hydrogen peroxide were found to have highly significant negative correlations with no fewer than three behavioral indicators in *C. elegans*. This led to the hypothesis that ACR may induce neurotoxicity by elevating oxidative stress levels in *C. elegans*, potentially related to *daf-16* and *skn-1*. Furthermore, GST functions as a double-edged sword in living organisms, triggering apoptosis while also inhibiting it and mitigating redox stress [70]. GST plays a pivotal role in the detoxification process, transforming ACR into non-toxic metabolites [70,71]. Our findings revealed a significant reduction in GSH levels, corresponding to increased ACR concentrations in a dose-dependent manner (Figure 2D). 

Upon entering the human system, ACR is metabolized through the action of glutathione-S-transferase (GST), wherein it reacts with GSH to yield the non-toxic metabolite N-acetyl-2-carbamoyl-L-cysteine [72]. The rate of GSH depletion in the body increases with higher concentrations of nematode exposure. The gene *gst-4* is crucial for regulating the nematode GST enzyme [73] and plays an essential role in mitigating the oxidative stress caused by ACR. Our research demonstrated that at ACR concentrations of 250 mg/mL and 500 mg/mL, the expression levels of *gst-4* mRNA and GST-4::GFP were significantly upregulated, indicating a mobilization of the nematode’s metabolic pathways to counteract toxic stress when exposed to ACR.

**Figure 8:**
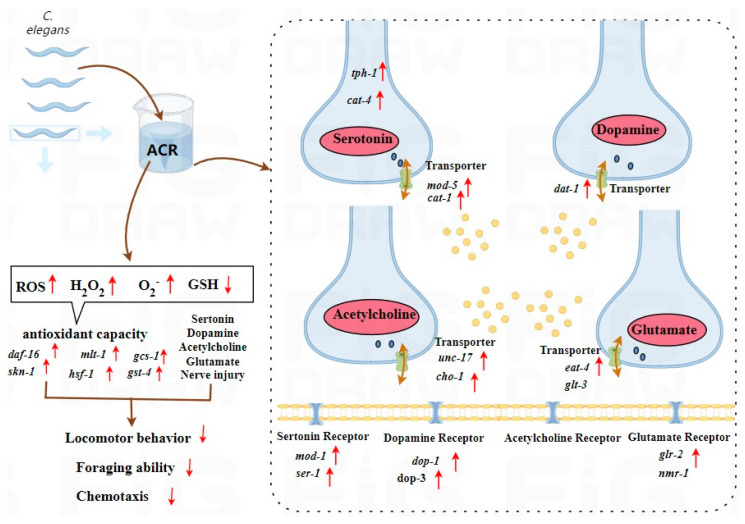
Diagram depicting the potential mechanism through which ACR may cause harm to serotonergic, dopaminergic, cholinergic, and glutamatergic neurons.

The behavioral impairments caused by ACR could stem from a complicated interaction between oxidative stress-related damage and irregular neurotransmission linked to neurotransmitters (Figure 8). Nevertheless, considering the complexities of the nervous system, additional research is needed to better understand the connection between neurotransmission and oxidative stress.

## 5. Conclusions

Our findings indicate that exposure to ACR adversely affects motor behavior, neuronal growth, neurotransmitter levels, and gene expression in nematodes. The neurotoxic effects of ACR are primarily attributed to abnormalities in neurotransmitter levels, as evidenced by significant elevations in serotonin, dopamine, acetylcholine, and glutamate, along with a notable upregulation of neurotransmitter-related genes, including *tph-1*, *cat-4*, *mod-1*, *mod-5*, *cat-1*, *ser-1*, *dat-1*, *dop-1*, *dop-3*, *unc-17*, *cho-1*, *eat-1*, and *glr-2*. Furthermore, ACR exposure resulted in increased levels of ROS, O_2_^−^, and H_2_O_2_ levels, while simultaneously decreasing GSH, which compromised the antioxidant system. This imbalance triggered a marked increase in the expression of nematode genes involved in the ACR detoxification pathway, such as *daf-16*, *skn-1*, *mlt-1*, *sod-3*, *gst-4*, *gcs-1*, *hsf-1*, and *hsp-16.2*. This study enhances our understanding of the potential neurobehavioral toxicity mechanisms of ACR and provides valuable information for health risk assessments related to ACR exposure. 

## Data Availability

Data are available on request from the authors.

## Supplementary Materials

The following supporting information can be downloaded at: https://www.mdpi.com/article/10.3390/antiox14060641/s1. Table S1: Primers for genes examined in this study (act-1 used as a reference gene).
