# Supplementary material for: Acrylamide Neurotoxicity Studies in Caenorhabditis elegans Model
Source: Antioxidants (Basel). 2025 May 27;14(6):641. doi: 10.3390/antiox14060641 (PMC12189583; doi:10.3390/antiox14060641)
Supplement: Supplementary file 1 [file antioxidants-14-00641-s001.zip › antioxidants-3597452-supplementary.pdf]

Supplementary Materials

Acrylamide Neurotoxicity Studies in Caenorhabditis Elegans Model

Zhonglian Ma [1,2, 3] ,Liang Ma [1,*] , Yuhao Zhang [1]

[1]College of Food Science, Southwest University, Chongqing, China

[2] College of Agronomy and Life Sciences Zhaotong University, Zhaotong, China

[3]Yunnan Key Laboratory of Gastrodia and Fungi Symbiotic Biology, Zhaotong University, Zhaotong, China

***Corresponding author**

Email: zhyhml@swu.edu.cn

Table S1. Primers for genes examined in this study (*act-1* used as a reference gene)

Gene	Forward primer	Reverse primer
tph-1	CTGCCGATTCTCCAGTAAAA	ACTACCCTCAACGGCATGTT
cat-4	TCGGAGAAGACATCAATCG	GCTCACAAAGGGAGAACATT
mod-1	TGGATGTGTGGATGCTTGGATGC	CATTCGTTCCCGTCGTCGTTCC
mod-5	CGCCGTCACTACCTCCATCATTA	GCGTCGTGTTCTCCAACTACC
cat-1	CGGTAGAAACTGAAGAACCTG	AGGCATAGTGTAGCCGATTC
ser-1	CCAGACGCTTCTCACCTCATCAAC	GCCGTGGAAGTGGTCATCATGTC
dat-1	ACCCAAAGATCCAGCAAGAGAACAG	CCAAATCTACAGCAAACCCGACAAC
dop-1	AGAGTTTTCGGATGCTGTTGGGAAG	CGCCGTCGTTCTATATTGTCAGGAG
dop-3	GGTGCCCAGATCGCTAGACAAATG	TCTTCAGACGATTCCGACATGGTTG
unc-17	ACCATCACAACCTGGATGTCCGAA	TCCATAGCCAACCCAACCATAGCA
cho-1	TGGGTTGGCGGTGCTTATA	TGGCTGCTGTCCAGAATGTTT
eat-4	TCTTATTAGCCAGTCTTATTCAC	GACCATTCTTCCTCCTCTT
glt-3	CGTTGCCAGTAACATTCC	TTCCGTCCATTGTAATTGTG
glr-2	TCTCTTCATACACGGCTAAT	CCTTCATTGACACCATACAG
nmr-1	GGAGATAATCGTCTGGAATTG	AGTGTATATGCTGATGATGTAAC
daf-16	GGAGCCAAGAAGAGGATAAAGG	GGAGAAACACGAGACGACGAT
daf-2	GTTCGCTGACAATCTCATTTGT	CAGTAATTTCACGTAGATGCGG
skn-1	TTCGCCTTCTCTCGAGGATATC	AACGTCTGCAAATCACATTCGT
mtl-1	AAGTACTGCTGTGAGGAGGC	GTTCCCTGGTGTTGATGGGT
sod-3	AGCATCATGCCACCTACGTGA	CACCACCATTGAATTTCAGCG
ctl-2	TCCCAGATGGGTACCGTCAT	GGTCCGAAGAGGCAAGTTGA
gst-4	TCAATGAGTCTCCAACGAGGAATCC	GAACCAGCCCGTGATGATTTCTTG
gcs-1	GTCGATGAAGCCAGATGGTTGT	CGATCGTCGACACTTGCACTAA
hsf-1	TTGACGACGACAAGCTTCCAGT	AAAGCTTGCACCAGAATCATCCC
hsp-16.2	TGAAGCGCCAAAGAAAGAAGC	TCAAGTTTATTGCAGCGAACAAT
act-1	CATGAAGATCAAGATCATCGCC	GTGACGATGGTTTTGAACTTGT
